# Outer-coordination sphere in multi-H^+^/multi-e^–^molecular electrocatalysis

**DOI:** 10.1016/j.isci.2021.103628

**Published:** 2021-12-15

**Authors:** Soumalya Sinha, Caroline K. Williams, Jianbing “Jimmy” Jiang

**Affiliations:** 1 Department of Chemistry, University of Cincinnati, P.O. Box 210172, Cincinnati, OH 45221, USA

**Keywords:** Catalysis, Electrochemistry, Electrochemical energy production, Electrochemical materials science

## Abstract

Electrocatalysis is an indispensable technique for small-molecule transformations, which are essential for the sustainability of society. Electrocatalysis utilizes electricity as an energy source for chemical reactions. Hydrogen is considered the “fuel for the future,” and designing electrocatalysts for hydrogen production has thus become critical. Furthermore, fuel cells are promising energy solutions that require robust electrocatalysts for key fuel cell reactions such as the interconversion of oxygen to water. Concerns regarding the rising concentration of atmospheric carbon dioxide have prompted the search for CO_2_ conversion methods. One promising approach is the electrochemical conversion of CO_2_ into commodity chemicals and/or liquid fuels, but such chemistry is highly energy demanding because of the thermodynamic stability of CO_2_. All of the above-mentioned electrocatalytic processes rely on the selective input of multiple protons (H^+^) and electrons (e^–^) to yield the desired products. Biological enzymes evolved in nature to perform such redox catalysis and have inspired the design of catalysts at the molecular and atomic levels. While it is synthetically challenging to mimic the exact biological environment, incorporating functional outer coordination spheres into molecular catalysts has shown promise for advancing multi-H^+^ and multi-e^–^ electrocatalysis. From this Perspective, herein, catalysts with outer coordination sphere(s) are selected as the inspiration for developing new catalysts, particularly for the reductive conversion of H^+^, O_2_, and CO_2_, which are highly relevant to sustainability. The recent progress in electrocatalysis and opportunities to explore beyond the second coordination sphere are also emphasized.

## Graphical abstract

**Figure:**
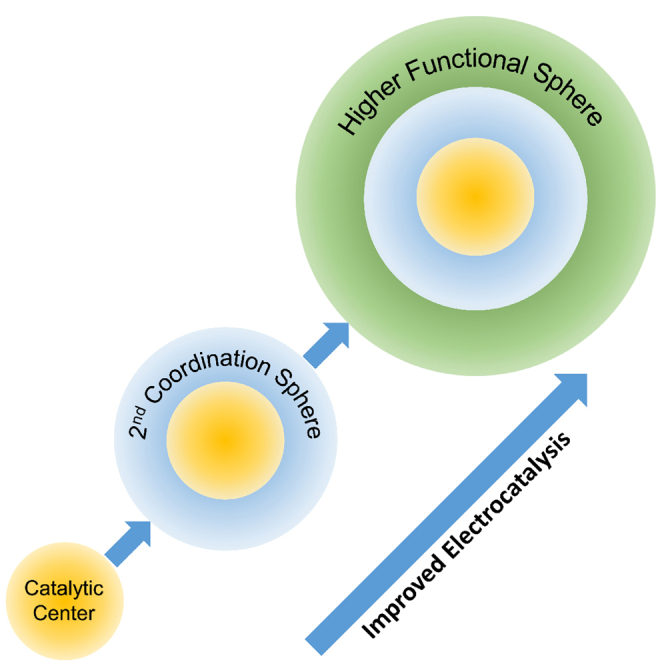

## Introduction

Increasing energy and environmental concerns have prompted the search for alternative energy resources. Despite the remarkable progress achieved in the past decades, the commercialization of clean and sustainable energy technologies is still limited(Lewis and Nocera, 2006). Hydrogen gas is an attractive fuel owing to its high enthalpy of combustion (−143 kJ/mol), and is a primary feedstock for fuel cells(Lewis, 2007; Turner, 2004). However, the industrial-scale production of H_2_ still depends on metallic Pt or its alloys, highlighting the need for efficient and inexpensive catalysts(Winter and Brodd, 2004). Thus, exploring robust catalysts comprising earth-abundant elements has become immensely important for producing H_2_ or performing the key fuel cell reactions.

The rapidly increasing atmospheric CO_2_ level (at present >410 ppm) due to the combustion of carbon-based fuels is alarming and requires urgent attention(Ballantyne et al., 2012). Although CO_2_ reduction chemistry began in the 1940s, steady accomplishments in this research area began in the late 1970s, followed by the confluence of climate concerns early in this century(Keith et al., 2018; Robert, 2016). The fundamental research goal is to capture atmospheric CO_2_ and convert it into commodity chemicals such as methanol, formic acid, ethylene, and carbon monoxide(Boot-Handford et al., 2014; Centi and Perathoner, 2009). These transformations require selective delivery of both protons (H^+^) and electrons (e^–^) to generate specific products(Robert, 2016).

Progress in the development of electrocatalysts to promote multi-H^+^ and multi-e^–^ transfer reactions has been accomplished during the last 40 years, yet the challenges in the organization of proton and electron inventories remain the same, which is a fundamental requirement for achieving product selectivity and catalytic stability. To facilitate ideal electron and mass transport, biological systems, such as metalloproteins, employ highly optimized chemical cascades to drive multi-H^+^ and multi-e^–^ transfer processes that include secondary sphere effects. A few examples of such enzymes are: (1) hydrogenases, which catalyze reversible H_2_ evolution reactions in aqueous media at or near thermodynamic potential(Madden et al., 2012; Shafaat, 2021), (2) cytochrome *c* oxidase (C*c*O), which selectively reduces O_2_ with 4H^+^/4e^–^(Kaila et al., 2010; Wikström et al., 2015), and (3) carbon monoxide dehydrogenases (CODH), which reversibly catalyze the reduction of CO_2_ to CO(Svetlitchnyi et al., 2001). Thus, such enzymatic mechanisms have inspired the study of redox reactions in molecular electrocatalysis where the selective coupling of H^+^ and e^–^ is the central focus(Amanullah et al., 2021). Although establishing a bioidentical environment in synthetic models is challenging, research efforts to incorporate nature’s features into molecular electrocatalysts have afforded promising results(Amanullah et al., 2021; Dey et al., 2017). In this context, the concept of a second coordination sphere integrated with the catalyst’s active site is the state-of-the-art approach. This Perspective emphasizes the application of the outer coordination sphere in electrocatalysis in which synthetic catalysts mimic the feature(s) at the active site of enzymes. The present discussion is limited to three examples of energy-related electrocatalysis, the H_2_ evolution reaction (HER), O_2_ reduction reaction (ORR), and CO_2_ reduction reaction (CO_2_RR), with a primary focus on how second coordination spheres direct multi-e^–^/multi-H^+^ transfer and promote chemical transformations. Although there are many reviews on the HER(Amanullah et al., 2021; McKone et al., 2014), ORR(Machan, 2020; Pegis et al., 2018), and CO_2_RR(Francke et al., 2018; Robert et al., 2020), herein, electrocatalysts with second and higher coordination spheres that mimic the function of biological motifs are highlighted, thus inspiring novel molecular designs for energy-related electrocatalysis. We also extend the discussion to include the effects of the higher coordination sphere as a promising strategy for enhancing the catalytic efficiency that includes kinetic and thermodynamic aspects. A list of the standard thermodynamic potentials for some selective multi-e^–^/multi-H^+^ reactions of interest is presented in Table 1 that will guide to estimate the overpotential for the electrocatalysts discussed later in this Perspective.

**Table 1:** Thermodynamic potentials of the electrochemical reactions of interest

Reactions	Standard potential^a^ (vs. NHE)	Standard potential^b^ (vs. Cp_2_Fe^+/0^)
2H^+^ + 2e^–^→ H_2_	0.00 V	−0.28
O_2_ + 4e^–^ + 4H^+^→ 2H_2_O	+1.23 V	+1.21
CO_2_ + 2e^–^ + 2H^+^→ CO + H_2_O	−0.10 V	−0.12
CO_2_ + 2e^–^ + 2H^+^→ HCOOH	−0.20 V	−0.84^c^

a Reported at pH 0.(Machan, 2020; Pegis et al., 2015; Zhao et al., 2020).

b Reported in MeCN.(Pegis et al., 2015).

c Calculated based on the conversion factor of −0.64 V vs Cp_2_Fe^+/0^.(Pegis et al., 2015).

## H_2_ Evolution Reactions

The prime objective of the hydrogen economy is achieving large-scale H_2_ production from water as an energy source. However, the challenge for H_2_ production by electrocatalysis is to achieve a high turnover frequency (TOF) at low overpotential using earth-abundant catalysts. In nature, hydrogenases in many microorganisms such as algae and bacteria couple 2H^+^ and 2e^–^ to produce H_2_, or oxidize H_2_ molecules with a TOF of up to 10^4^ mol/s per mole of the enzyme in the aqueous medium(Thauer et al., 2010). Although immobilizing enzymes as thin films on electrodes has proven to be a viable strategy, the catalytic activity is not satisfactory (2–4 mA per 1 mM hydrogenase)(Vincent et al., 2007). Therefore, designing molecular HER catalysts that mimic the structural features found in nature remains an ongoing active research focus.

### Enzymatic strategies

**Figure 1:**
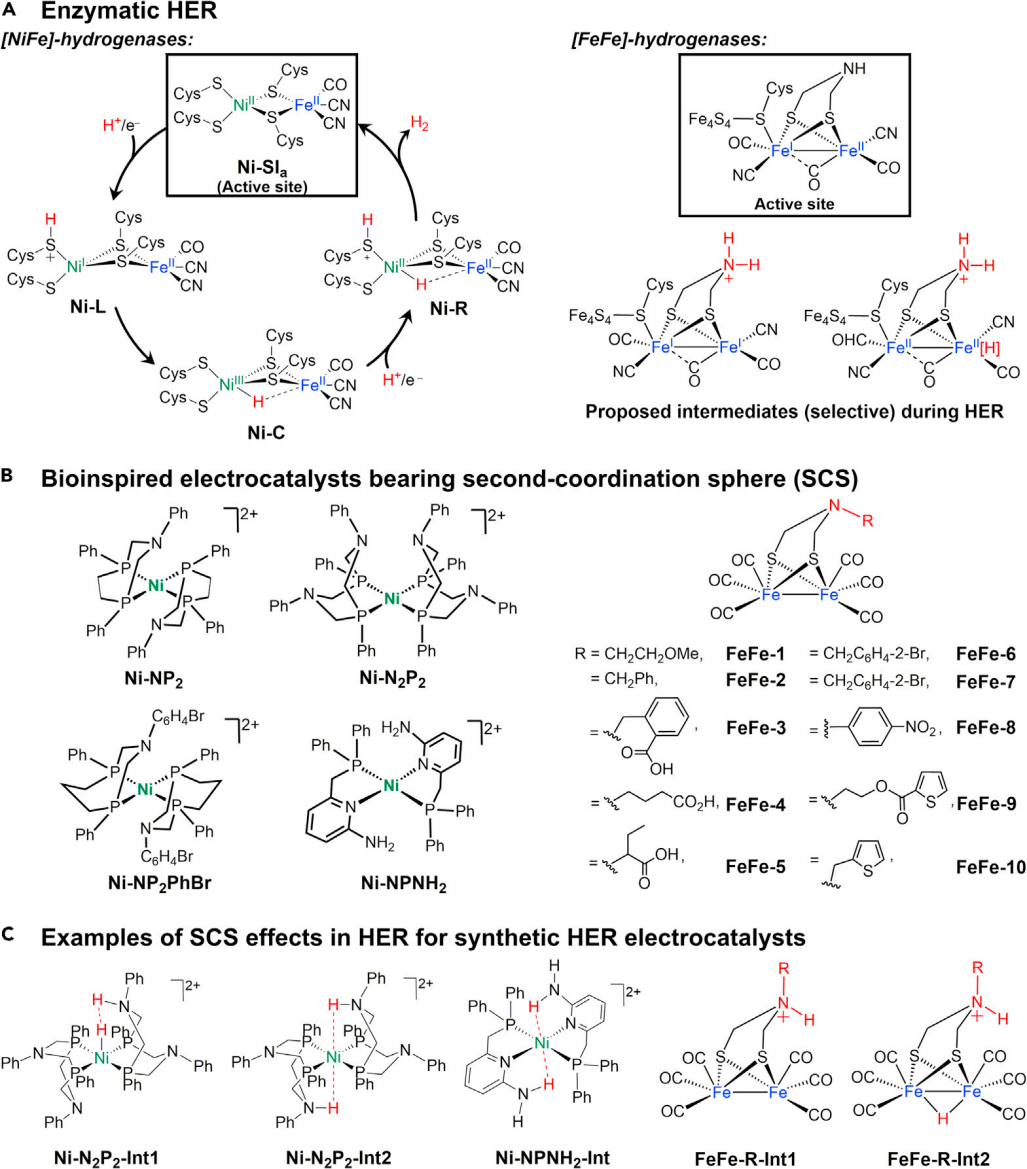
Enzymatic and synthetic HER models (A) [NiFe]-hydrogenases and [FeFe]-hydrogenases known for enzymatic HER(Shafaat, 2021). (B) Selected bio-inspired molecular HER electrocatalysts bearing second-coordination sphere (SCS). (C) Two proposed intermediates (**Ni-N**_**2**_**P**_**2**_**-Int1/Int2**) for **Ni-N**_**2**_**P**_**2**_, one intermediate (**Ni-NPNH**_**2**_**-Int**) for **Ni-NPNH**_**2**_, and two intermediates (**FeFe-R-Int1/Int2**; R groups are shown in (B)), showing different intermediate stabilization effects of SCS in H^+^ transfer events during HER(Helm et al., 2011; Tatematsu et al., 2016).

Three types of hydrogenases catalyze the HER: [NiFe]-hydrogenases,[FeFe]-hydrogenases, and [Fe]-hydrogenases(Fontecilla-Camps et al., 2007; Lubitz et al., 2014). Herein, we highlight the mechanisms by which the first two types, ([NiFe]-hydrogenases and [FeFe]-hydrogenases), which are most common in nature, catalyze the HER. First-row transition metals (Ni and Fe) form the active sites of [NiFe]-hydrogenases. The metals are bridged by two cysteine (Cys) thiolates (Figure 1A). Additionally, the Ni center is ligated with two other terminal Cys groups that direct H^+^ transfer while promoting the interconversion of H^+^ and H_2_(Lubitz et al., 2014; Shafaat, 2021). It is proposed that four intermediate species, **Ni-SI**_**a**_, **Ni-L**, **Ni-C**, and **Ni-R** (Figure 1A), are generated by [NiFe]-hydrogenases during the HER (identical intermediates are generated during H_2_ oxidation)(Shafaat, 2021). The coordinated Cys thiolate groups at the Ni center systematically control H^+^ delivery, with consequent H_2_ evolution at nearly zero overpotential. Notably, the three FeS clusters connect the active site of the [NiFe]-hydrogenases to the surface of the enzyme surface and facilitate multi-electron transfer events for the HER.

The FeS cluster in [FeFe]-hydrogenases plays similar roles in redox processes, but the proton transfer steps for proton relay are controlled by an additional secondary amine unit from the second coordination sphere (Figure 1A).(Amanullah et al., 2021) [FeFe]-hydrogenase catalyzes the HER in a more complicated manner than [NiFe]-hydrogenase(Amanullah et al., 2021). The key intermediate for [FeFe]-hydrogenases is Fe(I)Fe(I) with a protonated bridgehead N atom (Figure 1A). The ensuing protonation step involves electron transfer from the FeS cluster to form a terminal Fe(II)Fe(II)-hydride (H) species (Figure 1A) that releases H_2_ upon the addition of a proton. Thus, the [FeFe]-hydrogenases deliver H^+^ based on the presence of a bridgehead amine functionality in the second coordination sphere during the HER.

### Bioinspired electrocatalysts for HER

Since the crystallographic data for [NiFe]-hydrogenase were initially reported in 1995, the synthesis of biomimetic models using synthetic inorganic and organometallic tools has been attempted(Ahmed and Dey, 2019; Simmons et al., 2014; Tard and Pickett, 2009). However, only a few synthetic models can mimic the structure–function relationship observed in the enzymatic cycle(Brazzolotto et al., 2016). Although modeling the synthesis of bimetallic complexes containing Ni and Fe or other metals (e.g., Co, Mn, Ru, etc.) remains challenging, a considerable number of bioinspired mononuclear Ni electrocatalysts bearing proton donor(s) in the second coordination sphere have been reported, and have demonstrated remarkable performance for homogeneous electrocatalysis in organic media(Bullock and Helm, 2015; Helm et al., 2011; Wiese et al., 2013). Herein, we highlight a few examples from the library of electrocatalysts for the HER that incorporate pendant Brønsted base(s) in the outer-coordination sphere. Dubois, Helm, and others reported diphosphine-chelating Ni electrocatalysts, **Ni-NP**_**2**_, **Ni-N**_**2**_**P**_**2**_, and **Ni-NP**_**2**_**PhBr** (Figure 1B), which utilize flexible amine functionalities in the second coordination spheres to shuttle H^+^, as well as stabilize the Ni-hydride species (**Ni-N**_**2**_**P**_**2**_**-Int1** and **Ni-N**_**2**_**P**_**2**_**-Int2**) during the HER (Figure 1C).(Helm et al., 2011; Rountree and Dempsey, 2015; Wiese et al., 2013) Thus, the roles of these flexible arms in the outer coordination sphere resemble the function of Cys in [NiFe] hydrogenases. These molecular Ni electrocatalysts exhibit high turnover frequencies (TOF, 3,300–100,000 s^−1^, Table 2) for the HER in strong acids such as protonated dimethylformamide ([(DMF)H]^+^ (p*K*_a_ = 6.1))(McCarthy et al., 2014), and anilinium (p*K*_a_ = 10.6)(McCarthy et al., 2014) in wet (>1 M H_2_O) MeCN(Helm et al., 2011; Rountree and Dempsey, 2015). Furthermore, Masuda et al. reported a Ni^II^ electrocatalyst (**Ni-NPNH**_**2**_, Figure 1B) with a comparatively high TOF of 8,400 s^−1^ (Table 2) for H_2_ evolution from a weak acid, acetic acid (p*K*_a_ = 23.5) (Tatematsu et al., 2016). This catalyst employs a phosphinopyridyl ligand with amine groups in the second coordination sphere to stabilize the Ni-hydride intermediate, **Ni-NPNH**_**2**_**-Int** (Figure 1C). Overall, these HER electrocatalysts showed stable catalytic currents over the duration of controlled potential electrolysis and the production of H_2_ was confirmed using gas chromatographic analysis.

**Table 2:** Selective electrocatalysts^a^and their activity for HER

Catalysts	TOF (s^−1^)	Overpotential (V)	Solvent	Proton source
Ni-NP_2_	106, 000	0.72	MeCN	[(DMF)H]^+^ + H_2_O
Ni-N_2_P_2_	5670	∼0.3^b^	MeCN	CF_3_CO_2_H
	590	∼0.4^b^	MeCN	[(DMF)H]^+^
Ni-NP_2_PhBr	3300	0.76	MeCN	[(DMF)H]^+^ + H_2_O
Ni-NPNH_2_	8400	0.59	MeCN	CH_3_CO_2_H

a For **FeFe-x,** x = 1–10, see (Amanullah et al., 2021).

b Calculated based on the reported data. Overpotential = standard potential (corrected considering the p*K*_a_ of the acid used) – applied potential.

[FeFe]-hydrogenases also inspired the investigation of binuclear Fe complexes as molecular HER electrocatalysts. However, the commonly synthesized binuclear Fe complexes include six CO ligands (Figure 1B) and both Fe centers are expected to be reduced to Fe(0) before protonation. These binuclear synthetic hexacarbonyl Fe complexes (**FeFe-x**, x = 1–10, Figure 1B) contain an amine unit with a variety of R-substituents as a Brønsted base. The Brønsted base in the second coordination sphere is protonated in the presence of a strong acid, and thus resembles the function of the bridgehead amine in [FeFe]-hydrogenases (Amanullah et al., 2021).

**Figure 2:**
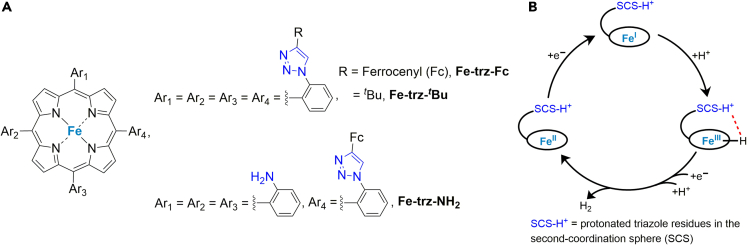
Examples of iron-porphyrins for HER (A) Iron-porphyrins bearing triazole units in the second coordination sphere. (B) Proposed overall HER mechanism for the complexes in (A), derived from the original work reported by Dey et al(Amanullah et al., 2021). The black circle around Fe represents the porphyrin rings.

The use of the pendant base in the second coordination sphere to control H^+^ transfer in the HER is not limited to bioinspired Ni-based electrocatalysts. Iron-porphyrins bearing distal triazole residues in the outer sphere (**Fe-trz-Fc**, **Fe-trz-**^***t***^**Bu**, Figure 2A) have also shown promise for catalyzing the HER in organic and aqueous media(Rana et al., 2017). These iron-porphyrins catalyze the HER at the Fe(I) center, and the catalytic currents increase upon the addition of acid. Notably, density functional theory (DFT) calculations supported the proposed mechanism for the HER using these iron-porphyrins, where the Fe(I)-center forms Fe^III^–hydride species upon protonation and the protonated triazoles in the second coordination sphere stabilize such intermediates through dihydrogen bond formation (Figure 2B). The red dotted bond in Figure 2B indicates stabilization of the H-bond between the protonated triazole residues and Fe^III^-hydride species. Thus, such second coordination spheres in iron-porphyrins provide thermodynamic advantages in the HER by increasing the affinity of H^+^ for the metal-hydride intermediate(Liu et al., 2012).

## ORR

Electrochemical reduction of O_2_ with 4H^+^/4e^–^ for H_2_O production is an important reaction in fuel cells and secondary metal-O_2_ batteries(Behling, 2013). Water must be selectively produced via the ORR in the fuel cell and in metal-O_2_ batteries as other partially reduced oxygen species, such as the 2H^+^/2e^–^ reduction product, H_2_O_2_(Borup et al., 2007), could harm the membrane separators. Therefore, the development of ORR electrocatalysts capable of selective O_2_-to-H_2_O conversion near the thermodynamic potential has gained significant attention in the last five decades(Machan, 2020). Although there are numerous review articles dealing with ORR electrocatalysis(Dey et al., 2017; Pegis et al., 2018), we herein focus on bioinspired electrocatalysts possessing second-coordination spheres for achieving enhanced catalytic activity and stability, and reduced overpotential.

### Biological ORR strategies

**Figure 3:**
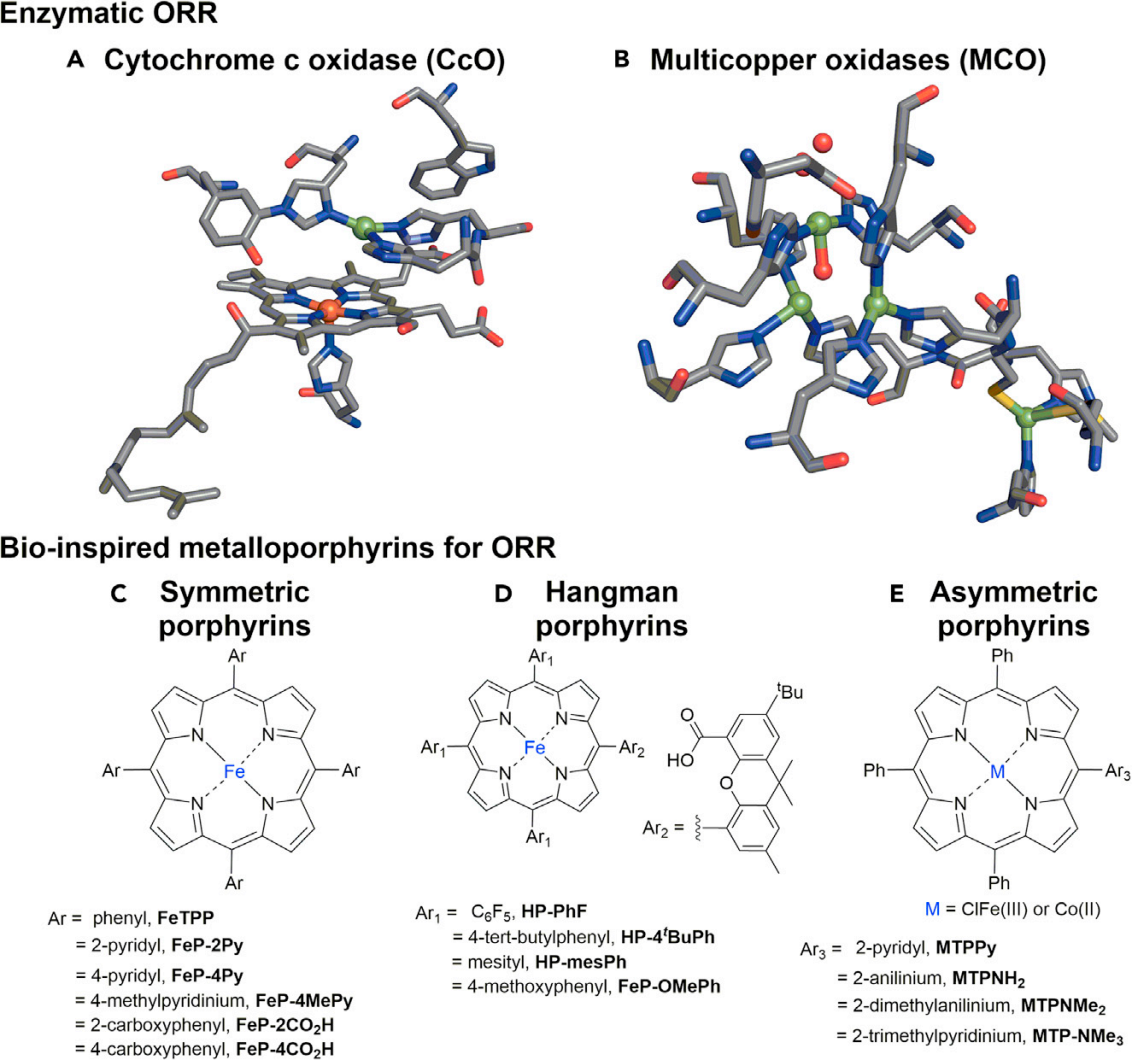
Enzymatic and synthetic ORR models Active site of (A) cytochrome *c* oxidases and (B) multicopper oxidases. The figures are adapted from the work reported by Dey et al.(Dey et al., 2017). (C) Symmetric Fe-porphyrins, (D) Hangman porphyrins, and (E) asymmetric metalloporphyrins bearing proton relays(Dey et al., 2017).

Nature has optimized metalloproteins, such as C*c*O, to favor the 4H^+^/4e^–^ reduction of O_2_(Kaila et al., 2010; Wikström et al., 2015), in which the active sites comprise an Fe-containing heme (heme a_3_), a distal Cu, and a tyrosine amino acid (tyrosine 244, Figure 3A); these species control the delivery of electrons and protons to ensure the selective reduction of O_2_. The heme *a*_3_ is an iron porphyrin that putatively generates a (Fe^IV^ = O)-porphyrin^•+^ complex during catalysis(Dey et al., 2017). The Cu site and tyrosine244 supply electrons, and proton delivery is managed by a transmembrane H-bonding network that also supports the essential proton-pumping activity of C*c*O(Yoshikawa and Shimada, 2015). Thus, the ORR selectivity is controlled by sequential H^+^/e^–^ transfer events. Multicopper oxidases (MCOs) are another example of O_2_-reducing metalloenzymes that have multiple redox centers (one type 1, one type 2, and two type 3 Cu centers) at the active center (Figure 3B).(Solomon et al., 1996) The mechanism of the ORR in MCOs preferentially follows the concerted 4e^–^/4H^+^ reduction of O_2_ to H_2_O(Solomon et al., 2014). The oxidation states of copper alternate between Cu^I^ and Cu^II^ to provide the required 4e^–^. Impressively, some MCOs facilitate the ORR at nearly the thermodynamic potential of the O_2_/H_2_O couple(Cracknell and Blanford, 2012). Overall, such structure–function relationships at the active sites of C*c*O and MCO provide strong motivation for the design of molecular ORR electrocatalysts.

### Bioinspired ORR electrocatalysts

Designing bioinspired metalloporphyrins for selective 4H^+^ and 4e^–^ transfer and evaluating their performance are contemporary research topics. In this context, Iron tetraphenylporphyrin (**FeTPP**), a simple synthetic model of C*c*O, provides a remarkable electrocatalytic platform for the homogeneous ORR(Pegis et al., 2018). Modifying the **FeTPP** ring at the *meso*-positions using weak Brønsted bases or acids such as 4-pyridyl (**FeP-4Py**)(Matson et al., 2012) and 4-carboxyphenyl (**FeP-4CO**_**2**_**H,** Figures 3C) and 3(Carver et al., 2012) respectively, afforded improved ORR activity compared to **FeTPP** under homogeneous conditions. The catalytic ORR activity was further improved by introducing 2-pyridyl (**FeP-2Py**)(Matson et al., 2012) or 2-carboxyphenyl (**FeP-2CO**_**2**_**H**)(Carver et al., 2012) substituents at the *meso*-positions of the **FeTPP** ring. The effects of the position of pendant proton relays in the second-coordination sphere (Figure 3C) on the selectivity and kinetics of O_2_ to H_2_O conversion were compared (Table 3).(Dey et al., 2017) Hangman catalysts are another remarkable example of asymmetric porphyrins (Figure 3D), where one *meso*-position carries a dibenzofuran moiety that is further connected to a -CO_2_H unit to enable proton relay(Chang et al., 2003). The presence of the single proton relay enhanced the ORR activity and selectivity toward H_2_O formation(Rosenthal et al., 2007).

**Table 3:** Selective ORR electrocatalysts^a^ and their activity for selective O_2_-to-H_2_O conversion

Catalysts	TOF (s^−1^)	Overpotential (V)	Solvent	Proton source
FeTPP	40,000	0.8	DMF	Et_3_NH^+^
FeP-2CO_2_H	200	0.4	MeCN	[(DMF)H]^+^
FeP-2Py	600	∼0.8^b^	aqueous (pH 0)	HOTf
FeTPPy	7840	∼1.0^b^	aqueous (pH 0)	H_2_SO_4_
CoTPPy	15,960	∼0.8^b^	aqueous (pH 0)	H_2_SO_4_
CoTPNMe_3_	1430	∼1.0^b^	aqueous (pH 0)	H_2_SO_4_
	1183	∼0.8^b^	aqueous (pH 7)	H_2_SO_4_

a For more examples, see (Dey et al., 2017; Machan, 2020; Pegis et al., 2018).

b Estimated based on the reported data as described in Table 2.

Asymmetric metalloporphyrins bearing second-coordination spheres have also been immobilized onto graphite electrode surfaces for heterogeneous ORR in aqueous electrolytes(Sinha et al., 2015, 2019). The objective is to study proton relay on solid electrode surfaces and how molecules with pendant proton relay behave on carbon surfaces during the ORR in an aqueous solution. For example, **FeTPPy** (Figure 3E) bearing a single pyridyl group showed stable catalytic currents over 5 h of controlled-potential electrolysis (CPE) in O_2_-saturated 1 M H_2_SO_4_ (pH 0), whereas the catalytic current of the parent **FeTPP** declined after 15 min of CPE under identical electrochemical conditions(Sinha et al., 2015). Additionally, both **FeTPP** and **FeTPPy** immobilized on edge plane graphite showed similar Faradaic efficiencies of ∼90% toward H_2_O production(Sinha et al., 2019). Notably, the inclusion of a single pyridyl group in **FeTPPy** at the *meso*-position did not affect the overpotential compared to that of **FeTPP** for O_2_-to-H_2_O conversion at pH 0.

Furthermore, replacing the Fe(III) center in **FeTPPy** with Co(II) yielded another ORR electrocatalyst, **CoTPPy**, which catalyzed the ORR with selective H_2_O production, without compromising the overpotential and catalytic stability. The effect of a single pyridyl group in changing the selectivity of Co-porphyrins is remarkable, as most Co-porphyrins, including **CoTPP**, promote the 2H^+^/2e^–^ reduction, leading to H_2_O_2_ production, which is undesirable for fuel cells(Sinha et al., 2019). Furthermore, electrostatic stabilization of the Co-oxo intermediate during the ORR using the cationic ancillary group -NMe_3_^+^ in the second coordination sphere of **CoTPNMe**_**3**_ also afforded pH-dependent selectivity toward H_2_O formation (Figure 3E).(Zhang and Warren, 2020) The above-mentioned examples validate the high applicability of second coordination spheres in enhancing the catalyst stability and selectivity for the ORR.

## CO_2_RR

Converting anthropogenic CO_2_ into sustainable liquid fuels (methanol, methane, formic acid, etc.), or into fuel precursors such as syngas (H_2_ and CO), to yield hydrocarbons has gained immense attention in recent research trends(Robert, 2016). Electrochemical conversion is a very promising approach for reducing CO_2_ via multi-H^+^/multi-e^–^ steps using electrocatalysts, including metal electrodes such as Cu, Sn, Au, or Pd (Cui et al., 2020; Godbold et al., 2021; Robert et al., 2020). Although heterogeneous CO_2_RR models are highly suitable for industrial-scale CO_2_ reduction, molecular CO_2_RR electrocatalysts are desirable for elucidating the fundamental CO_2_RR mechanism. In electrochemical CO_2_RR catalysis, the electron-rich electrode surface supplies multiple electrons for the reaction, but multi-H^+^ transfer often compromises the product selectivity. Typically, the metal center of inorganic catalysts first gets reduced upon e^–^ transfer and follow the protonation-first pathway that forms a metal-hydride which reacts with another H^+^ to release H_2_, or with CO_2_ to yield formate. Alternatively, the reduced metal center can undergo CO_2_-activation-first pathway and selectively produces CO upon sequential H^+^ transfer events(Barlow and Yang, 2019). The Sabatier principle states that such catalyst-substrate interaction is significant in catalysis and the related free-energy should be favorable(Ooka et al., 2021). Furthermore, DuBois, Yang, Berben, and many others emphasized the hydricity of the metal hydride species that determine the free energy for the reaction between a metal hydride and H^+^ or CO_2_ and thus, controls the product selectivity(Barlow and Yang, 2019; Loewen and Berben, 2019; Rakowski Dubois and Dubois, 2009). However, similar thermodynamic landscape in CO_2_RR also can be established by incorporating proton donors into the outer-coordination sphere of molecular electrocatalysts for supplying H^+^ during the CO_2_RR(Costentin and Savéant, 2017). Additionally, chemical modification beyond the active site of molecular CO_2_RR electrocatalysts offers advantages, such as (1) higher activity by stabilizing the CO_2_ bound intermediate, (2) higher activity provided by proton-rich environments (i.e., proton relays and hydration spheres), and (3) optimization of the selectivity for the CO_2_ reduction reaction.

### Biological CO_2_RR strategies

**Figure 4:**
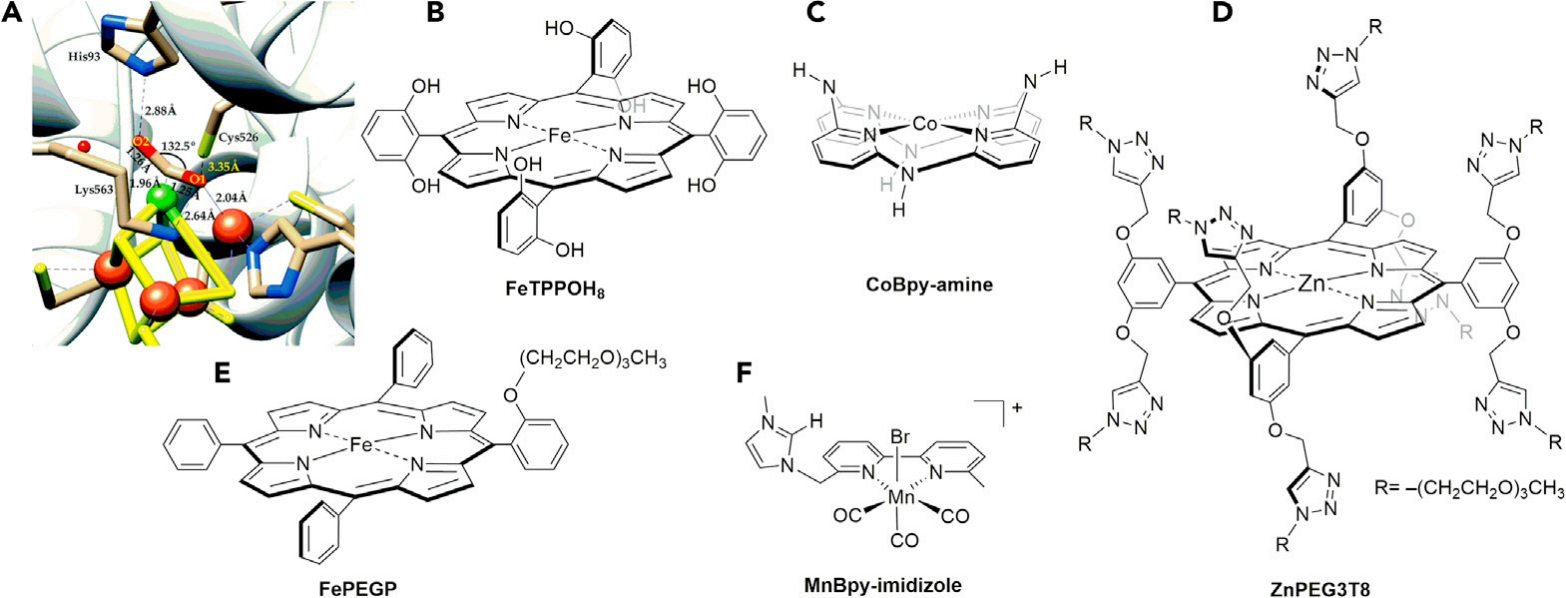
Enzymatic and synthetic CO_2_RR models (A) Active site of [NiFe]CO dehydrogenases(Amanullah et al., 2021), (B) Fe-porphyrin bearing eight pendant proton relays (**FeTPPOH**_**8**_)(Costentin et al., 2012), (C) Co-bipyridine with a bridging pendant amine group (**CoBpy-amine**)(Chapovetsky et al., 2018), (D) Zn-porphyrin containing 8 triazole units each with PEG proton relays(Williams et al., 2020), (E) Fe-porphyrin with a single PEG proton relay(Chaturvedi et al., 2021), (F) Mn-bipyridine with imidazole in the second coordination sphere(Sung et al., 2019).

Enzymes such as [NiFe]CO dehydrogenases ([NiFe]CODHs) are natural substances that catalyze the CO_2_ to CO interconversion reaction(Mondal et al., 2015). The mechanism of CO_2_RR catalysis by these enzymes is still under investigation or complicated(Jeoung and Dobbek, 2007). It is generally proposed that CO_2_ binds at the Ni center, and the Fe center coordinates with an O atom of CO_2_. Interestingly, the same O atom forms an H-bond with a nearby lysine (Lys563, Figure 4A), and the other O atom of CO_2_ forms an H-bond with a proximal protonated histidine (His 93, Figure 4A). Upon binding with the Ni center, the angle of the C-O-C bond in CO_2_ is proposed to be 132.5°, and the C–O bond is elongated to 1.26 Å due to charge transfer to the CO_2_ antibonding orbitals via back-bonding(Amanullah et al., 2021). Overall, this is viewed as the enzymatic activation of CO_2_ to CO_2_^•–^, where the proximal amino acids (Lys563 and His93) stabilize the intermediate through H-bonding. Thus, such biological CO_2_RR catalysts motivate the design of artificial catalysts incorporating pendant outer-sphere residues. In this Perspective, we selectively chose some bioinspired electrocatalysts (Figure 4) that use the outer coordination spheres for the selective CO_2_-to-CO conversion. If there are any such electrocatalysts that produce major products other than CO, we noted in our discussion.

### Intermediate stabilization in molecular electrocatalysts

Stabilization of the bound substrate is critical for enhancing CO_2_ reduction. Hydrogen bonding and electrostatic interactions effectively stabilize the [catalyst-CO_2_^•–^] adduct upon CO_2_ binding at the reduced metal center, thereby minimizing the energy barrier (ΔG^#^) or overpotential for CO_2_RR electrocatalysis(Costentin and Savéant, 2017). The **FeTPPOH**_**8**_ electrocatalyst (Figure 4B) developed by Savéant et al. possesses a local proton source within the catalyst, serving as a remarkable example of enhanced catalytic activity due to sphere-assisted catalysis by functional groups in the second coordination sphere(Costentin et al., 2012). Such pendant phenolic groups in close proximity to the active site facilitate rapid protonation of the substrate, allowing for a lower overpotential and higher activity compared to iron porphyrins that do not have acidic groups in the second coordination sphere. In a study of cobalt complexes (Figure 4C), Marinescu et al. found that the pendant amine unit stabilizes the cobalt/carboxylate intermediate, and the number of pendant amines is directly proportional to the rate of catalytic CO_2_ reduction(Chapovetsky et al., 2018). As a critical aspect, the study demonstrated a non-cooperative effect between the amine units in the higher coordination sphere, suggesting that each amine is individually involved in catalysis. DFT calculations reveal that the amine groups do not directly interact, but rather associate, with acid molecules to provide protons for stabilizing the intermediate(Chapovetsky et al., 2018).

### Proton relays

Because the CO_2_ reduction pathway favors proton-coupled electron transfer (PCET) steps, proton relays continuously furnish protons to the active site to enhance the catalytic activity. To mimic the complex enzymatic system found in nature, simpler proton relays have been designed for CO_2_ reduction. Several hydrophilic groups have been utilized to achieve this shuttling effect, including phenol(Margarit et al., 2018; Nichols et al., 2020; Sinha and Warren, 2018), triazole(Lashgari et al., 2020; Sen et al., 2019), polyethylene glycol (PEG)(Chaturvedi et al., 2021; Lashgari et al., 2020), imidazole(Sung et al., 2019), urea(Gotico et al., 2020; Haviv et al., 2018), and amine(Jakobsen et al., 2021). The relay can be as short as a single OH group in the second coordination sphere, as achieved using an asymmetric Fe-porphyrin carrying a single *ortho*-phenolic group at the *meso*-position and three phenyl groups at the remaining *meso*-positions of the Fe-porphyrin ring(Sinha and Warren, 2018). Such asymmetric iron complex remained stable for approximately 2 h at −2.3 V vs. Fc^+/0^ after an initial drop in current. Our group observed that a triazole bundle attached to a zinc porphyrin complex (**ZnPEG3T8**, Figure 4D) afforded improved catalytic activity by enabling enhanced proton relays(Lashgari et al., 2020). Another example is an iron porphyrin with a single PEG chain in the second coordination sphere (Figure 4E).(Chaturvedi et al., 2021) It was demonstrated that proton relays do not have to completely create a “hydration sphere”, as observed in many previous successful systems(Gotico et al., 2019; Roy et al., 2017). Imidazolium groups can also act as a proton shuttle. Nippe et al. designed and studied a manganese bipyridine complex (Figure 4F), where imidazolium groups created a hydration shell around the active site(Roy et al., 2017; Sung et al., 2019). The increased presence of protons caused by hydrogen bonding networks surrounding the active site can improve catalysis by furnishing the protons required for CO_2_ reduction.

### Selectivity enhancements

**Figure 5:**
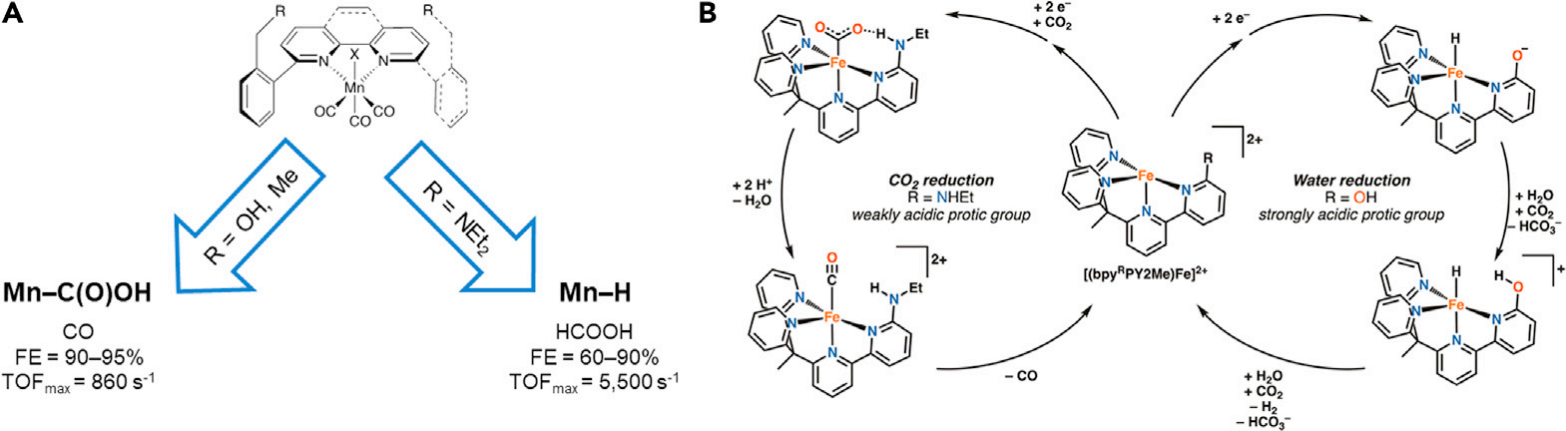
Selectivity in CO_2_RR (A) Schematic depicting selectivity differences of a Mn catalyst with different groups in the second coordination sphere(Rønne et al., 2020). (B) Two competing catalytic pathways for the catalytic reduction of CO_2_ to CO or water to H_2_ by non-heme iron complexes(Zee et al., 2020). Adapted with permission from (Zee et al., 2020). Copyright 2020 American Chemistry Society.

Achieving product selectivity in the CO_2_ reduction reaction is a continuing challenge because of the large number of possible products (CO, formate, methanol, etc.), for which the thermodynamic potentials are very similar(Kuhl et al., 2012). Accomplishing the desired selectivity is one of the major obstacles hindering the progress of CO_2_ electroreduction. The selectivity for CO_2_ reduction is governed by the formation of different catalytic intermediates, which can be selectivity altered by the secondary coordination sphere's effect on the p*K*_a_ of the catalytic active site. A manganese bipyridine complex with different pendant groups is one example in which the selectivity is enhanced by modifying the second coordination ligand structure (Figure 5).(Rønne et al., 2020) When amine units were incorporated into the second coordination sphere, a Mn-hydride species was formed during catalysis, which is critical for formate production (Figure 5A). Alternatively, when a hydroxyl or methyl group was attached to the second coordination sphere, CO_2_ was directly bound to the Mn center, and CO was the major product. The acidity of the environment surrounding the metal active site was modified by the ligand's second coordination sphere and therefore was able to be tuned toward a specific electrocatalytic product.

In addition to product selectivity within the realm of possible CO_2_RR products, hydrogen evolution also competes with CO_2_ reduction because of the favored thermodynamics of the former. Overcoming the thermodynamic preference of hydrogen evolution is of great interest in designing CO_2_RR electrocatalysts. In another selectivity study by Long et al., the ligand choice in the second coordination sphere altered the catalytic pathway completely(Zee et al., 2020). With a strong protic unit such as OH, the iron non-heme catalyst favors the formation of the iron-hydride intermediate and therefore promotes water reduction. In comparison, when a weaker acid (–NHEt) is present in the second coordination sphere, the same iron active site preferentially forms the metallocarboxylate intermediate, leading to the reduction of CO_2_ to CO (Figure 5B). Both cases suggest that the second coordination sphere can influence the preferred intermediate species, ultimately impacting the product selectivity. Finally, we noted the performance of the CO_2_RR electrocatalysts discussed in this Perspective and tabulated the kinetic and thermodynamic parameters that are associated with their CO_2_RR reactivity in organic solvents (Table 4).

**Table 4:** Selective electrocatalysts and their activity for selective CO_2_RR

Catalysts	TOF (s^−1^)	Overpotential (V)	Solvent	Proton source
FeTPPOH8	208,000^a^	0.465	DMF	H_2_O
CoBpy-amine	16,900	–	DMF	TFE
FePEGP	140,000	–	MeCN	H_2_O
Mn-Bpy-imidizole	–	–	MeCN	H_2_O
ZnPEGT8	1,843	0.63^a^	DMF	H_2_O
MnBpy	5,500	0.60	MeCN	TFE
Fe-pyd	2,067	–	MeCN	H_2_O

a Estimated based on the reported data as described in Table 2.

## Beyond second-coordination sphere

**Figure 6:**
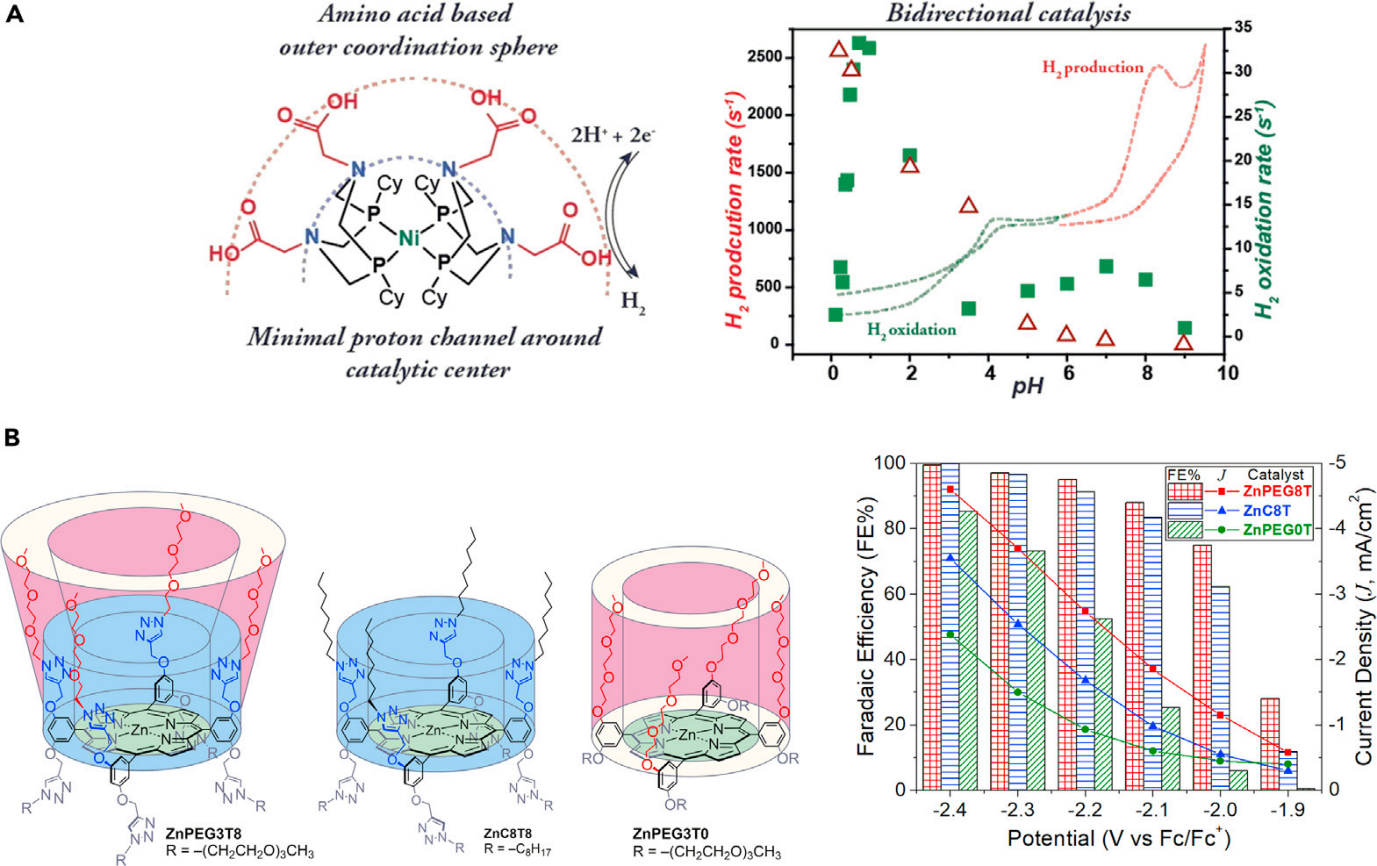
Higher coordination spheres (A) Schematic depicting the second and outer coordination spheres of a nickel diphosphine electrocatalyst for reversible hydrogen evolution(Dutta et al., 2013). (B) Schematic depicting the coordination sphere environments of **ZnPEG3T8**, **ZnC8T8**, and **ZnPEG3T0** with CO Faradaic efficiencies (FE%) and current densities (J) at various potentials for all compounds(Williams et al., 2020).

Coordination sphere effects can extend beyond those of the second coordination sphere. Higher coordination spheres enhance the catalytic efficiency by altering the surrounding chemical environment of the second coordination sphere. Shaw et. studied a nickel diphosphine HER catalyst with an amine-pendant phosphine ligand in the second coordination sphere and amino acid in the higher coordination sphere (Figure 6A).(Dutta et al., 2013) The higher coordination sphere acts as a proton shuttle for the proton relay to the second coordination sphere, providing an enzymatic effect. Our group studied the effects of PEG units in the higher coordination sphere, in conjunction with eight proton-donating triazole groups in the second coordination sphere (**ZnPEG3T8** in Figure 6B).(Williams et al., 2020) The catalytic activity of **ZnPEG3T8** was 30-fold higher than that of the control compound with alkyl chains in the higher coordination sphere (**ZnC8T8**). Another control experiment using a PEGylated, triazole-free complex (**ZnPEG3T0**) also showed inferior lysis efficiency, suggesting the cooperative effects of the second and higher coordination spheres. These examples demonstrate the long-distance effects of higher coordination spheres on catalytic performance, expanding the versatility of molecular designs.

## Outlook and future directions

Designing ligand frameworks with outer-sphere coordination is an attractive strategy for developing bioinspired molecular electrocatalysts capable of multi-H^+^ and multi-e^–^ transfer. Such strategies also help establish the Sabatier principle(Ooka et al., 2021) by stabilizing the catalyst–substrate intermediates through substituent effects for homogeneous electrocatalysis. These effects include H-bonding, the acidity of the catalytic site, and through-space electrostatic effects. Stabilization of these intermediates lowers the energy barrier for product formation, thereby lowering the overpotential for electrocatalysis. However, there is generally a trade-off between lower overpotentials and the TOF. Therefore, incorporating multiple outer sphere effects in molecular electrocatalysts is promising for boosting kinetic factors with minimal compromise of the overpotential.

Although a library of impressive homogeneous electrocatalysts bearing outer coordination sphere(s) for multi-H^+^ and multi-e^–^ transfer catalysis has been documented, practical energy devices to address global energy challenges demand heterogeneous electrocatalysts. Therefore, the immobilization of molecular electrocatalysts on solid electrodes is an emerging trend in heterogeneous electrocatalysis(Brunner et al., 2020; Geer et al., 2021; Sinha et al., 2020; Zhang and Warren, 2020). The major challenge in developing these systems is optimizing the synthetic strategies for immobilizing molecules on the electrode surface. Conjugation of molecules on the electrode surface through covalent linkages(Jackson et al., 2019; Kaminsky et al., 2019) or non-covalent adsorption(Brunner et al., 2020; Maurin and Robert, 2016; Sinha et al., 2019) are ongoing strategies for developing heterogeneous electrocatalysts, where selectively controlling the number of H^+^ and e^–^ transfers is of central interest. Nevertheless, the molecular features, including the outer-sphere effects, are often complicated by the electrode properties upon immobilization of the molecules. This is primarily because the catalytic sites reside within the electric double-layer (EDL)(Jackson et al., 2018). We propose that higher coordination spheres can increase the distance between the catalytic site and the electrode surface to facilitate electron tunneling across the EDL.

Despite significant current challenges in the practical development of electrocatalytic devices for multi-H^+^ and multi-e^–^ transfer catalysis on the industrial scale, the outer coordination sphere concept can facilitate the design and understanding of such catalysts to afford benchmark molecular electrocatalysts. Furthermore, the selectivity for multi-H^+^/multi-e^–^ redox reactions can also be enhanced through higher coordination sphere effects due to steric effects and changes in the p*K*_a_. This in turn changes the major reduction product by limiting the reactivity of the intermediate species (i.e., metal hydrides). Thus, a holistic approach for catalyst design that extends beyond the second coordination sphere, and focuses on higher coordination sphere effects will push the field of electrocatalysis closer toward understanding enzymatic catalysis. Fine-tuning large ligand scaffolds for electrocatalysts is expected to further drive this progress.
